# Estimated Intakes of Nutrients and Polyphenols in Participants Completing the MaPLE Randomised Controlled Trial and Its Relevance for the Future Development of Dietary Guidelines for the Older Subjects

**DOI:** 10.3390/nu12082458

**Published:** 2020-08-15

**Authors:** Daniela Martini, Stefano Bernardi, Cristian Del Bo’, Nicole Hidalgo Liberona, Raul Zamora-Ros, Massimiliano Tucci, Antonio Cherubini, Marisa Porrini, Giorgio Gargari, Raúl González-Domínguez, Gregorio Peron, Benjamin Kirkup, Paul A. Kroon, Cristina Andres-Lacueva, Simone Guglielmetti, Patrizia Riso

**Affiliations:** 1 Department of Food, Environmental and Nutritional Sciences (DeFENS), Università degli Studi di Milano, 20133 Milan, Italy; daniela.martini@unimi.it (D.M.); stefano.bernardi@unimi.it (S.B.); cristian.delbo@unimi.it (C.D.B.); massimiliano.tucci@unimi.it (M.T.); marisa.porrini@unimi.it (M.P.); gargari.g@gmail.com (G.G.); simone.guglielmetti@unimi.it (S.G.); 2 Biomarkers and Nutrimetabolomics Laboratory, Department of Nutrition, Food Sciences and Gastronomy, XaRTA, INSA, Faculty of Pharmacy and Food Sciences, University of Barcelona, 08028 Barcelona, Spain; n.hidalgoliberona@ub.edu (N.H.L.); rzamora@idibell.cat (R.Z.-R.); raul.gonzalez@ub.edu (R.G.-D.); gregorio.peron@ub.edu (G.P.); candres@ub.edu (C.A.-L.); 3 CIBER de Fragilidad y Envejecimiento Saludable (CIBERfes), Instituto de Salud Carlos III, 08028 Barcelona, Spain; 4 Unit of Nutrition and Cancer, Cancer Epidemiology Research Programme, Catalan Institute of Oncology (ICO), Bellvitge Biomedical Research Institute (IDIBELL), 08908 L’Hospitalet de Llobregat, Spain; 5 Geriatria, Accettazione Geriatrica e Centro di ricerca per l’invecchiamento, IRCCS INRCA, 60127 Ancona, Italy; a.cherubini@inrca.it; 6 Quadram Institute Bioscience, Norwich Research Park, Norwich NR4 7UG, UK; benjamin.kirkup@quadram.ac.uk (B.K.); paul.kroon@quadram.ac.uk (P.A.K.)

**Keywords:** nursing home, residential care, aging, menu, flavonoids, phenolic acids

## Abstract

The evaluation of food intake in older subjects is crucial in order to be able to verify adherence to nutritional recommendations. In this context, estimation of the intake of specific dietary bioactives, such as polyphenols, although particularly challenging, is necessary to plan possible intervention strategies to increase their intake. The aims of the present study were to: (i) evaluate the nutritional composition of dietary menus provided in a residential care setting; (ii) estimate the actual intake of nutrients and polyphenols in a group of older subjects participating in the MaPLE study; and (iii) investigate the impact of an eight-week polyphenol-rich dietary pattern, compared to an eight-week control diet, on overall nutrient and polyphenol intake in older participants. The menus served to the participants provided ~770 mg per day of total polyphenols on average with small variations between seasons. The analysis of real consumption, measured using weighed food diaries, demonstrated a lower nutrient (~20%) and polyphenol intake (~15%) compared to that provided by the menus. The feasibility of dietary patterns that enable an increase in polyphenol intake with putative health benefits for age-related conditions is discussed, with a perspective to developing dietary guidelines for this target population.

## 1. Introduction

It is well recognized that nutrition plays an important role in health status, with increasing evidence of associations between intake of specific dietary components and risk of many non-communicable diseases (NCDs), such as cardiovascular diseases (CVDs), type 2 diabetes, and some types of cancer. For instance, the Global Burden of Diseases has recently indicated that high intake of sodium, low intake of whole grains, and low intake of fruits are the leading dietary risk factors for deaths and disability-adjusted life-years (DALYs) worldwide [1]. These findings have been widely used to prepare national and international dietary guidelines aimed both at recommending the adequate intake of energy and nutrients for different targets of population and possibly at reducing the risk for the most common NCDs [2].

The ageing process affects the nutrient needs of older subjects, whose requirements for some nutrients may be reduced or increased with respect to younger adults. In this life-stage, a variety of factors such as sensory losses, chewing and swallowing problems, and medications may compromise dietary intake and lead to nutritional deficiencies and malnutrition, which has been contributing to the progression of many diseases and common syndromes in older people [3].

For this reason, specific recommendations have been proposed to meet the nutritional requirements of this target group; for instance, energy, protein and fibre intake should be individually adjusted by considering their nutritional status and physical condition and accounting for the presence of specific disease [4]. In addition to macronutrients, micronutrients also play a fundamental role in promoting health and preventing NCDs and their deficiencies are often common in aged people for a number of reasons including reduced food intake or lack of a varied diet, but they are also associated with the vicious cycle promoted by diseases and pharmacological treatments.

It is noteworthy that these factors may also affect the intake, absorption and/or metabolism of bioactive compounds such as polyphenols. In this regard, data on polyphenol intake in different older target groups are not univocal, possibly due to differences in geographical area considered, and in the individual characteristics in terms of health/disease status, and living conditions, as previously evidenced [5]. The interest in the assessment of polyphenol intake and the study of their potential impact on older subjects has been growing by considering several findings suggesting the protective role they can play against age-related diseases and in the promotion of healthy aging [6]. Regarding the changes on polyphenol intake with age, conflicting results have been reported so far, with some studies showing an increased intake [7,8] while others reporting no differences depending on age [9,10].

For the above-mentioned reasons, the nutritional assessment of older people represents a critical issue, which may be particularly true for those living in residential care settings where the prevalence of malnutrition has been reported to be extremely variable, ranging from 1.5 to 66.5% [11]. This represents a current clinical and public health concern at both the individual and population level [12,13]. Several methods have been developed for the assessment of energy and nutrient intake, including food-frequency questionnaires, food diaries and 24-h dietary recalls, all having pros and cons to be considered when choosing the best method to use in each specific context [14]. The estimation of micronutrients and bioactives like polyphenols is particularly challenging, mainly due to methodological issues, including the tool and the database used for the evaluation, as well as the type of polyphenol under consideration (e.g., total polyphenols versus single classes and subclasses of polyphenols) [5]. Being able to make accurate estimates of actual polyphenol intake is a fundamental requirement of developing a better understand of the role of these compounds and their relationship with health or disease conditions. In addition, this information is crucial to define potential polyphenol exploitation for the development of dietary strategies to prevent against age-associated diseases. 

Based on these premises, the aim of this research was to evaluate the nutritional composition of nursing home dietary menus and to estimate the actual intake of nutrients and polyphenols in a group of older subjects living in a residential care setting. The assessments were performed as part of the MaPLE (Microbiome mAnipulation through Polyphenols for managing Leakiness in the Elderly) project, funded within the European Joint Programming Initiative “A Healthy Diet for a Healthy Life” (JPI HDHL), with the aim to investigate benefits of a polyphenol-enriched diet on intestinal permeability in older subjects. An increased gut permeability, often associated with dysbiosis and inflammation, could play a role in the development of some age-related conditions. In this regard, it has been suggested that the intake of polyphenols may represent a promising strategy to improve intestinal permeability (IP) as demonstrated mainly in experimental studies suggesting the involvement of these bioactives in both direct and indirect modulatory mechanisms [15]. In this context, a more accurate estimation of the intake of polyphenols in a vulnerable target such as older subjects, in terms of amount, sources and distribution across the day and even in different seasons, can be of relevance. This could enable a better understanding of their potential benefits and the development of specific recommendations based on findings from dietary intervention studies.

## 2. Materials and Methods 

### 2.1. Study Design and Population

The study design of the MaPLE randomized controlled trial (RCT) has been previously reported [16]. Briefly, the central hypothesis that this study sought to address was that a polyphenol-enriched dietary pattern would reduce IP and systemic inflammation and cause beneficial changes in various biomarkers of cardiometabolic health, and that this would be associated with changes in the gut microbiota in these older subjects. To this aim, volunteers were randomized to consume a polyphenol-rich diet (PR-diet) or a control diet (C-diet) for 8 weeks following a cross-over design separated by an 8-week wash-out. The development of the PR-diet and C-diet has been reported previously [16]. During the intervention, subjects were given three small portions of polyphenol-rich foods daily as substitutes for foods with lower polyphenol contents that were part of the C-diet (developed by analyzing the regular menus provided to the study participants and specifically assessing the nutrient and polyphenol composition). The characteristics and polyphenol content of the servings provided in the PR-diet for each product are reported in Table 1. The amount of polyphenols provided was more than double that deriving from the replaced products. Data shown include total polyphenol content (i.e., TPC) quantified by analysing products through the Folin–Ciocalteau method [17] and estimates of total polyphenols (i.e., TP). The estimation of TP was calculated as the sum of flavonoids, phenolic acids, lignans, stilbenes and other polyphenol classes expressed in mg (aglycone/100 g). The estimations were performed using an in-house ad hoc database of food composition on polyphenols, compiled from the USDA (fdc.nal.usda.gov/) for databases (for flavonoids, isoflavones and proanthocyanidins) and the Phenol-Explorer (PE; www.phenol-explorer.eu) database (for phenolic compounds lignans, stilbenes and other minor polyphenol classes) through a computer application developed that uses the relational database system. This methodology has been used and previously described [18,19,20,21]. Polyphenols were expressed as mg of aglycones per 100 g.

For the intervention study, all the participants were recruited from residents at Civitas Vitae, a large residential care setting (OIC Foundation including both nursing homes and independent residencies for older subjects, Padua, Italy) according to specific inclusion and exclusion criteria. Among inclusion criteria, subjects had to be aged 60 years and to have increased intestinal permeability evaluated by serum zonulin level as previously reported [16].

All the participants recruited into the study were self-sufficient and were in good cognitive health. The Ethics Committee of the Università degli Studi di Milano approved the study protocol (15/02/2016; ref.: 6/16/CE_15.02.16_Verbale_All-7). All the participants were provided with detailed information about their involvement in the study and gave their informed consent before beginning the intervention. The trial was registered in the ISRCTN Registry on 28 April 2017; ISRCTN10214981.

### 2.2. Nutritional and Polyphenol Composition of the Menus 

To estimate the energy and nutrient composition of the planned meals regularly provided, the weekly menus during different seasons (summer, mid-season and winter) were evaluated (i.e., covering the whole intervention study). To this aim, Metadieta ^®^ software (Me.te.da srl, S. Benedetto del Tronto, Italy) was used to include all the recipes and to estimate the nutritional composition of the different menus.

In addition, the TPC content of the menus was estimated by PE databases with the addition of our own data (characterized products in Table 1 used for the intervention) and other literature sources for those ingredients that were not available in those databases [22,23,24]. TP was instead estimated through the PE/USDA database, as also described in Section 2.1.

### 2.3. Evaluation of Actual Energy, Nutrient and Polyphenol Intake 

During both intervention periods, weighed food records (WFR) were used to estimate food, energy, nutrient and polyphenol intake as reported in Section 2.2. In particular, up to six detailed daily diaries (recording the amount of foods provided and the amount actually consumed by weighing the leftovers) were analysed for each subject during the two intervention periods. In addition, one diary was filled in by participants at baseline and scheduled the day of blood drawings and sampling according to what was previously reported [16].

### 2.4. Statistical Analysis

Statistical analysis was conducted using the Statistical Package for Social Sciences software (IBM SPSS Statistics, Version 26.0, IBM corp., Chicago, IL, USA) and R statistical software (version 3.6.). One-way ANOVA was applied to analyse differences between the winter, mid-season and summer menus provided during the intervention in terms of nutrients and polyphenol composition. The nonparametric Wilcoxon–Mann–Whitney test with Benjamini–Hochberg correction pairing the data when possible was performed to ascertain differences at baseline between men and women in terms of actual intake and to verify the impact of treatment (PR vs. C-diet) and gender (men vs. women) on both nutrient and polyphenol intake in participants. The level of significance was set at *p* ≤ 0.05. All results were expressed as mean ± standard deviation (SD).

## 3. Results

Fifty-one older subjects (22 men; 29 women; age ≥ 60 y) successfully completed the entire study, and the data reported here are for those 51 participants. Dropouts were not due to side effects of the dietary intervention itself.

### 3.1. Nutritional Composition of Menus

The nutritional composition of the nursing home menus provided during the intervention is reported in Table 2. The average estimated daily energy content of the summer menu was 140 kcal higher than for the winter menu. No differences were detected for the nutrients among seasonal menus, both when expressed as net quantity or as percentage of energy provided.

**Figure 1:**
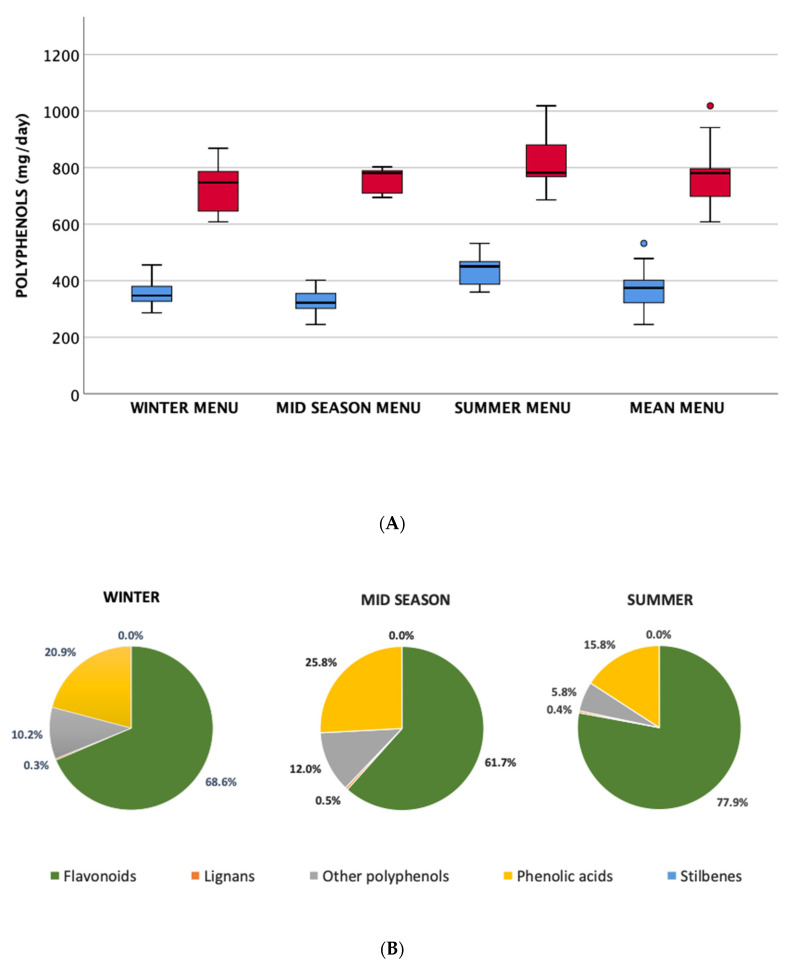
Box plot (panel **A**) showing polyphenol content in the seasonal menus, estimated through PE/USDA databases and other published data (TP in light blue) and by Folin–Ciocalteau data as reported in the PE database and other sources (TPC in red); percentage distribution of polyphenol classes (panel **B**) in the seasonal menus. Dots represent mild outliers that are more extreme than Q1 − 1.5 * IQR or Q3 + 1.5 * IQR but are not extreme data (where Q1=quartile 1; Q3=quartile 3; IQR=interquartile range).

Regarding the polyphenol composition of the menu, as shown in Figure 1, no significant differences were observed among the different seasonal menus, which had an estimated mean TPC of about 770 mg/day. 

### 3.2. Actual Energy, Nutrient and Polyphenol Intake at Baseline and during the Intervention

The actual energy, nutrient and polyphenol intake estimated at baseline for women, men and the whole group of participants is shown in Table 3. Overall, energy intakes, and accordingly nutrient intakes, were lower than calculated for the estimates based on the foods consumed from the menus, in keeping with the fact that not all the food was consumed for any particular meal. There were no significant differences between women and men for any of the dietary variables that were assessed at baseline. This was also confirmed by analysing the data obtained during the intervention study (Supplementary Materials Figure S1), except for simple carbohydrates in women and for total lipids and PUFA in men when comparing intake measured during the PR-diet and the C-diet (*p* < 0.05). Finally, differences were observed in ω-6 fatty acids, iron and calcium intake following the PR-diet in both women and men. 

Regarding polyphenols, flavonoids and phenolic acids were the most consumed classes and were comparable between women and men.

### 3.3. Polyphenol Intake at Baseline and during Intervention

**Figure 2:**
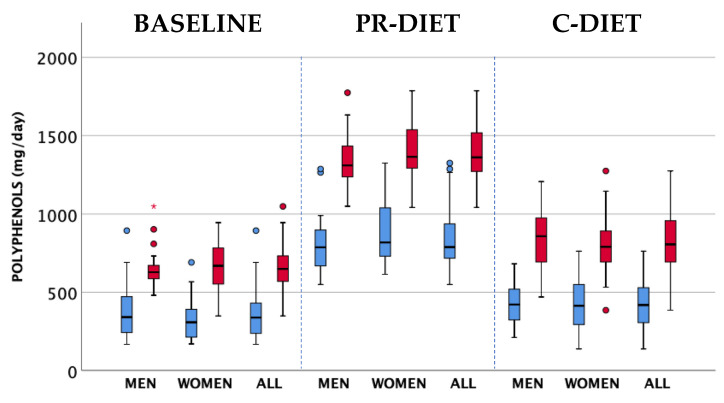
Polyphenol intake at baseline and in polyphenol (PR)-diet and control (C)-diet in the whole group of subjects and stratified by gender. The intake has been estimated using the PE and USDA databases and other published data (TP in light blue) and by Folin–Ciocalteau data as reported in the PE database and other sources (TPC in red). Dots represent mild outliers that are more extreme than Q1 − 1.5 * IQR or Q3 + 1.5 * IQR but are not extreme data. Asterisks are extreme data that are more extreme than Q1 - 3 * IQR or Q3 + 3 * IQR (where Q1=quartile 1; Q3=quartile 3; IQR=interquartile range).

Figure 2 shows the polyphenol intake at baseline and in the two intervention periods. At baseline, the intake of TPC was 663.4 ± 147.5 mg/d and comparable between women (669.2 ± 160.1 mg/d) and men (655.2 ± 130.8 mg/d). The consumption of PR-products significantly (*p* < 0.0001) increased the intake of TPC by about 600 mg/d compared to the C-diet and was comparable in both men and women.

Table 4 shows the contribution of different polyphenol classes to the total polyphenol intake during the PR and C-diet. Flavonoids were the main subclass increased in the PR-rich diet and accounted for 74.6%, followed by phenolic acids (23.3%), while lignans and other polyphenols accounted for the remainder. A treatment effect (*p* < 0.0001) for total flavonoids and phenolic acids was observed (Table 4), while a gender effect was observed for stilbenes showing a higher intake in men compared to women (*p* = 0.033).

**Figure 3:**
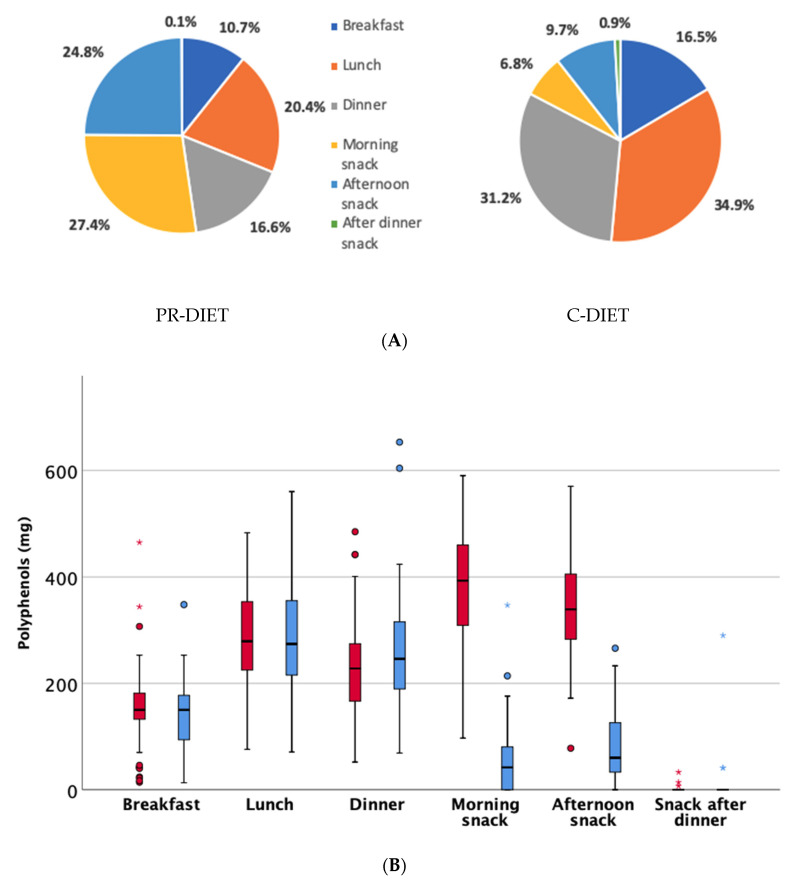
Total phenolic contribution from different meals during the polyphenol (PR)-diet and the control (C)-diet expressed as percentage (Panel **A**), or expressed as amount in mg during the PR-diet (in red) and the C-diet (in blue) as estimated through PE/USDA databases and other published data (Panel **B**). Dots represent mild outliers that are more extreme than Q1 − 1.5 * IQR or Q3 + 1.5 * IQR but are not extreme data. Asterisks are extreme data that are more extreme than Q1 − 3 * IQR or Q3 + 3 * IQR (where Q1=quartile 1; Q3=quartile 3; IQR=interquartile range).

Considering the total polyphenol (TP) contribution from the different meals, in the PR-diet, ~50% of polyphenol intake derives from snacks and the remaining ~50% from breakfast, lunch and dinner (Figure 3). In particular, there is a significant contribution to mid-morning and afternoon snacks from the intake of PR-products. Conversely, during the C-diet, only ~15% of the total polyphenols consumed were derived from snacks. 

Overall, through the analysis of the menu items provided to the volunteers and recorded in the WFRs during the two intervention periods and by considering the frequencies of consumption of the single ingredients, we estimated the main polyphenol sources contributing to the different meal times. During the PR-diet, the main foods providing polyphenols at breakfast were fruit and fruit-derived products (e.g., orange, grape, orange juice, apricot jam, etc.), followed by barley coffee and minor contributions from coffee and tea. Polyphenol-rich products on the PR-rich diet were occasionally consumed at breakfast, where green tea, pomegranate juice, chocolate callets and blood orange juice were the most commonly consumed. For lunch and dinner, the main sources during the PR-diet were vegetables (e.g., chard, asparagus, broccoli, carrots), extra virgin olive oil, legumes and spices. A few participants occasionally consumed white wine in small portions (usually 1 glass), which also made a contribution to the polyphenol intake. PR-rich products were mainly consumed as mid-morning and mid-afternoon snacks, as reported in Figure 3. During the C-diet, we found similar foods providing polyphenols at breakfast, lunch and dinner compared to the PR-diet, except for the introduced PR-rich products. Major differences between the two treatments were largely due to the snack foods because only fruits and fruit-based products (i.e., juices), cakes (including sometime chocolate-based cakes) or yogurt were available during the C-diet, whereas a more extensive range of PR-foods were available as snacks on the PR-rich diet.

## 4. Discussion

The evaluation of the adequacy of diets in older subjects is of utmost importance not only to identify possible deviations from desirable nutritional targets but also to contribute to the development of new recommendations that address gaps in the current guidance. In this context, the MaPLE project has given us the unique opportunity to assess dietary intake in a well-controlled setting where it is also possible to analyse the daily menus provided to the residents, while considering all the recipes and ingredients used for the preparation of the meals. At the same time, long-term residences often have facilities enabling the measurement of food intake (e.g., by collecting multiple weighed food records) and this represents the best procedure to estimate actual consumption. Menu planning in residential care involves modifications of recipes during the year to take account of seasonal changes in ingredient availability and this may partially affect not only nutritional characteristics in terms of macro- and micro- nutrients but also food sources of bioactive compounds with potential impact on host metabolism and other functions.

In the present study, the evaluation of three different menus showed that overall they were comparable in terms of nutritional composition, and also that they were in line with the dietary recommendations for older subjects in Italy (i.e., Italian Reference Intake) [2], with some dissimilarities that are worth highlighting. In regards to total energy, menus provided suitable amounts for the target population, at least in consideration of the main Italian guidelines developed for dietary management in residential care [25]. Some studies carried out in nursing homes showed lower energy provided by menus [26,27], while others reported data higher or similar to our observation [28,29,30]. The distribution in macronutrients was consistent with the recommendations: carbohydrates accounted for ~47% of total energy intake on average (reference intake range: 45–60% energy (E)), although we found there was a higher intake of simple carbohydrate in comparison with the recommendations (20% E vs. < 15% E) due to the wide use of fruit juices and hot beverages with added sugars as has been commonly reported in this target population. Protein intake derived mainly from animal sources (about two-thirds) and was higher in comparison with the suggested dietary target (1.1 g/kg/day), while total lipid intake was within the reference intake range (20–35% E). Specifically, SFAs were in accordance with the national/international recommendation (<10% E), while total PUFAs were slightly lower than 5% E due to the low intake of ω-6 in favour of higher MUFAs, as can often be found in the Mediterranean areas. The amount of fibre provided by the menus was slightly lower than the suggested dietary target of 25 g per day defined by Italian and international guidelines [2,31]. Regarding micronutrients, iron contribution was adequate while, as also reported in the literature, calcium content in the three menus was lower than the population reference intake (PRI, 1200 mg for both women and men ≥ 60 years) [2]. However, it is worth noting that these data included only calcium derived from recipes and did not consider contributions from other sources such as water and supplements. Vitamin B1, B6 and B12 provided by menus were higher than reference values, while folates were slightly lower than the established population reference intake of 400 μg per day. With regard to antioxidants, vitamins E and C were both adequate, in particular vitamin C largely exceeded the PRI levels (i.e., 85 mg and 105 mg per day for women and men respectively). Overall, the results on the nutritional composition of the menus suggest that, although they are generally developed following specific guidelines, it is still possible to improve the content of critical nutrients such as fibre, specific micronutrients and bioactives, above all in institutionalised subjects as also reported in the literature [29,30].

Notably, actual food intake in older subjects can be significantly lower with respect to that provided by the menus. For these reasons, we also estimated the actual food consumption through the analysis of detailed and repeated weighed food records. Measured energy and nutrient intake were indeed lower than that provided through the menus (by about 20%), with no differences between women and men. In this regard, it is underlined that the subjects enrolled in the present study generally had a good nutritional status, evidenced also by their anthropometric characteristics (BMI = 26.8 ± 5.5 kg/m^2^).

The energy intakes we have reported here (mean approximately 1580 kcal) were slightly lower than those found in the InCHIANTI study, performed on about 1200 free-living older subjects (>65 years) in Tuscany, in which mean energy intakes ranged from 1764 to 2260 kcal/d and from 1521 to 1793 kcal/d in men and women, respectively [32]. However, despite the higher energy intake, in the InCHIANTI study, a large group of subjects reported inadequate intakes of protein, calcium and other nutrients, which have been independently associated with frailty [33]. In our assessments, the lower food intake was associated with reduced protein intake (about 0.9 g/kg day on average), increasing the rate of inadequate intake above all in male subjects (about 22% with intake ≤ 0.71 g/kg per day and only 18% with intake ≥ 1.1 g/kg per day as defined by the suggested dietary target). The consumption of simple carbohydrates in older subjects was confirmed to be higher than the suggested values, while the fat intake appeared to be within the suggested intake range, although the amount of ω-6 fatty acids remained lower than recommended values, as did the intake of calcium, vitamins B1, B6 and folates. These results confirmed previous observations of a potential risk of long-term inadequate intake of nutrients that are fundamental for maintenance of functional and metabolic integrity in older subjects, and that these inadequate intakes are likely due to the actual food intake being significantly less than the amount of food provided to the care home residents in each meal (i.e., incomplete meal consumption is likely a major cause). Moreover, there is not only a problem related to overall food intake but also to specific classes of products that appear to be consumed in lower amounts with respect to others, for example justifying a low intake of fibre that has been found for most, if not all, the subjects under study. This is an underestimated consideration that should be a target for future multidisciplinary research that is able to finally implement guidelines for the achievement of nutritional targets through traditional or possibly alternative strategies.

A major focus in this study was polyphenols because these compounds have the potential to provide further specific benefits to the target population under study. It has been reported that there is a large variation in the polyphenol content of foods available in different periods of the year [34,35,36], and for this reason we specifically analysed recipes and ingredients used to develop seasonal menus and the results obtained showed a relatively comparable amount of these bioactive compounds (about 770 mg per day on average as TPC) among the different seasons. We could not find other data on the impact of seasonality on polyphenol content of dietary plans provided in long-term residences for older people, while more literature is available in free-living older subjects. In this regard, in the Blue Mountains Eye Study, a longitudinal study performed in Australia [35], the authors found that season did not affect the overall total flavonoid intake in a group of adult and older subjects; however, it was relatively higher in spring and lower in autumn in line with our results. Conversely, Tatsumi et al. [37] showed that total antioxidant intake in a Japanese population (39–77 years) was highest in winter and lowest in summer. The authors attributed this difference to the participants’ selection of food (in particular fruits and vegetables) but also beverages across seasons.

In our study, the assessment of actual food consumption at baseline indicated a mean TPC intake of ~660 mg/d (i.e., evaluated by Folin–Ciocalteau through the PE database and specific literature), about 15% less than the amount estimated in the menus served to the study participants. Although a thorough comparison with other published data must be done cautiously because of the differences in the populations under study and the methods and databases used for estimating the intakes of total polyphenols and polyphenol classes, the overall actual intake estimated in the present study seems to be comparable with mean intake observed in the InChianti study [20], but lower with respect to others previously reported. 

In fact, assessments in older subjects estimated polyphenol intakes from 333 mg/day up to 1492 mg/day, as reported previously [5]. For example, in the PREDIMED study evaluating a big cohort of Spanish older subjects aged 55–80 years, a mean polyphenol intake of 820 ± 323 mg/day expressed as glycosides was estimated through the PE database, by analysis of food consumption data obtained from FFQs [38]. With regard to the contribution of the classes, total flavonoid intake is generally the larger part of the intake, while data available in some studies suggest that up to 30–40% of the total polyphenol intake can be represented by phenolic acids [5]. Results from the EPIC cohort showed that older subjects tended to have increased intake of flavonoids, stilbenes, lignans, and other polyphenols with respect to younger individuals, while no differences were found for total polyphenol intake [7], and similar findings were reported by Karam and colleagues [8], also showing an impact of gender. In our study in a controlled setting, the data confirmed that the flavonoid subclass was the greatest contributor to total polyphenol intake followed by phenolic acids, while no differences were detected between men and women. Some studies have suggested a higher total and subclass polyphenol intake in females compared to males [8,10], above all when standardized by energy intake, and this may also be the reason for the lack of differences in our study. In addition, it is relevant that the overall lower availability of food alternatives for selection in controlled, with respect to a free-living condition, may have affected eating behaviour, increasing the comparability of the dietary intake.

With regard to polyphenol food sources, tea and coffee have been underlined as the main polyphenol contributors in northern European older subjects, while red wine, extra virgin olive oil and fruit are the main sources in Southern Europe [7,39]. In our evaluation, fruit and fruit juices, vegetable and extra virgin olive oil represent the main food categories providing polyphenols. In addition, we could not demonstrate a different selection of polyphenol sources depending on gender, despite some studies having reported a higher contribution from fruit and vegetables in females compared to males [8,34]. It is noteworthy that in the nursing home, the intake of coffee and wine was strongly limited, if not denied, to limit risks associated with caffeine and alcohol consumption and this may represent an important behavioural difference with respect to what may be observed in free-living older subjects.

The evaluation of habitual polyphenol intake in the older target group was a fundamental step in the process of developing a reliable and evidence based polyphenol rich dietary pattern to use for the intervention trial. In particular, the aim was to approximately double the habitual polyphenol intake of the nursing home residents when on the PR-rich diet in order to reach amounts in the highest quantile of intake identified in previous observational studies, where older subjects were included or specifically considered [7,21,40].

Indeed, the main objective of the MaPLE study was to investigate whether the increased intake of polyphenols might cause a reduction in intestinal permeability (IP) and inflammation associated with an improved intestinal microbial ecosystem, also affecting metabolic and functional activities in the older subjects [16]. In particular, the intervention was developed by replacing three portions per day of low polyphenol foods/beverages with specific products rich in polyphenols. The selection of the products was performed by considering different aspects: (i) the total amount of polyphenols provided, (ii) the contribution of the different polyphenol classes, (iii) the adequate portion of food able to provide a reliable high dose of polyphenols, and (iv) the possible food preparation in order to ensure polyphenol bioavailability. Additionally, foods selection was carried out by considering the characteristics of the target group and their specific needs in terms of acceptability and suitability in the context of residential care settings. Through the administration of the selected foods, we provided mainly flavonoids (approximately four times higher compared to the amount introduced through the C-diet) and phenolic acids. These bioactives have been suggested as potential modulators of critical factors and specific targets regulating IP, including the impact on microbiota composition and activities [15,41]. Overall, our results demonstrate that it is possible to obtain a significant increase in polyphenol intake in older subjects, through the use of small amounts of well-accepted polyphenol-rich food products. Moreover, it has been demonstrated that the intake is well tolerated and without undesirable effects. Participants appreciated the products and were interested in continuing with the dietary protocol after the end of the trial, suggesting that older people can change their diet if it does not dramatically modify their eating habits.

An interesting observation highlighted was that older subjects preferred the consumption of PR-products during the intervention as mid-morning and afternoon snacks. In fact, the protocol adopted did not fix the timing for the PR-food intake, but the products should have been consumed within the day according to preferences and/or habits. For this reason, our results give an important contribution to the development of dietary guidelines for this target population. At the same time, the analysis of the pattern of consumption of polyphenol-rich foods may also contribute to a better understanding of chronobiological aspects related to the effect of bioactive compounds. In this regard, it has been suggested that the inclusion of polyphenols within the meals may have an impact on related metabolic responses, e.g., through reduction of glucose and lipid levels, inflammation, oxidative stress, and blood pressure, associated with food intake [42,43,44]. Consuming most of the polyphenols outside of the main meals could also affect their bioavailability for direct absorption and their use as substrates for microbial transformation.

This work has several strengths mainly related to the well-controlled setting of the intervention, enabling both the evaluation of the nutrient and bioactive content of the menus and the actual intake during the whole intervention, ensuring high adherence to dietary instructions. Conversely, possible study limitations include the small sample size and the partial generalizability to free-living community dwelling older subjects. Finally, the limited food choices available in the main standard menus provided could have reduced the possibility of showing gender differences.

## 5. Conclusions and Perspectives

In conclusion, the assessments performed within the MaPLE project have further underlined the need for a careful revision of dietary menus addressed for older subjects not only to optimize the intake of essential nutrients, but also of bioactive compounds, such as polyphenols, in order to lower the risk of chronic diseases and improve specific metabolic and functional activities during aging. In this context, we have shown that there is a possibility to develop feasible and reliable polyphenol-rich dietary patterns that can be appreciated and consumed by the older population with excellent compliance, while assuring a significant increase in the intake of these bioactive compounds. Moreover, the products and preparations included in the dietary menu have been easily managed in the residential care setting and this is a practical aspect of relevance for the success of new recommendations.

Further studies are needed to: (i) improve tools available to better estimate polyphenol intake and enable comparison of different data in the literature, as previously reported [5]; and (ii) improve dietary recommendations by defining the amount of polyphenol needed in order to obtain, if confirmed, the postulated health benefits in the older subjects. This is not an easy task and imply a strong research effort that needs to consider the potential impact of these results for the development of evidence-based dietary guidelines for the management of age-related conditions.

## Figures and Tables

**Table 1:** Polyphenol content and composition of each serving of MaPLE products included in the dietary intervention, expressed as mg per serving.

	TPC	TP	Flavonoids	Phenolic Acids	Stilbenes	Lignans	Other
Blood orange juice	178	63.4	42.0	21.4	-	-	-
Blood orange fruit	178	34.8	23.1	11.8	-	-	-
Renetta apple	296	225.9	201.2	24.7	-	0.01	-
Renetta apple purée ^+^	167	150.6	134.1	16.5	-	0.00	-
Whole blueberry ^§^	291	259.5	165.1	94.5	-	-	-
Blueberry purée ^✥^	259	199.0	163.6	35.4	-	0.0	0.02
Pomegranate juice	189	135.5	55.1	80.3	-	-	-
Green tea *	146	129.2	116.2	13.0	-	0.08	-
Cocoa powder °	234	92.2	90.5	1.7	-	0.00	0.01
Chocolate callets	337	167.8	165.4	2.4	0.01	-	-

^§^ Frozen whole blueberry product was thawed and prepared before consumption; ^✥^ Blueberry purée was a ready-to-eat product; ° Cocoa powder was dissolved in hot milk or water; * Green tea was prepared by solubilization of 200 mg of green tea extract in 200 mL of hot water; ^+^ Renetta apple purée was prepared in controlled conditions and stored at −18 °C until consumption. TPC, total polyphenol content by Folin–Ciocalteau assay; TP, total polyphenols determined by USDA and Phenol Explorer databases.

**Table 2:** Mean energy and nutrient composition of the nursing home menus across three seasons and overall mean composition.

Nutritional Factor	Winter Menu	Mid-Season Menu	Summer Menu	Mean Menu
Energy (kcal)	1889 ± 102 ^a^	2012 ± 176 ^a,b^	2028 ± 66 ^b^	1976 ± 133
Total CHO (% of energy)	47.4 ± 3.2	46.4 ± 4.7	46.5 ± 3.0	46.8 ± 3.5
Simple CHO (% of energy)	20.6 ± 2.2	19.8 ± 0.6	20.3 ± 1.4	20.2 ± 1.5
Total protein (% of energy)	18.7 ± 2.5	20.0 ± 2.3	19.6 ± 2.7	19.4 ± 2.5
Animal protein (% of energy)	11.1 ± 2.8	13.4 ± 2.8	12.8 ± 2.5	12.4 ± 0.3
Plant protein (% of energy)	6.2 ± 0.9	6.2 ± 1.1	6.4 ± 0.8	6.3 ± 0.1
Total Lipids (% of energy)	34.1 ± 4.2	33.7 ± 4.1	34.0 ± 4.6	33.9 ± 4.1
SFA (% of energy)	8.7 ± 1.3	8.9 ± 2.0	8.6 ± 1.8	8.7 ± 1.6
MUFA (% of energy)	17.9 ± 3.3	16.9 ± 2.4	17.7 ± 2.2	17.5 ± 2.6
PUFA (% of energy)	3.7 ± 0.8	3.8 ± 0.7	3.9 ± 1.4	3.8 ± 1.0
ω-3 (% of energy)	0.7 ± 0.4	0.7 ± 0.4	0.7 ± 0.4	0.7 ± 0.4
ω-6 (% of energy)	3.0 ± 0.9	3.0 ± 0.8	3.2 ± 1.3	3.0 ± 1.0
Total Fibre (g/1000 Kcal)	12.2 ± 2.1	11.6 ± 2.4	12.3 ± 1.7	12.0 ± 2.0
Cholesterol (mg)	264 ± 91	358 ± 134	288 ± 123	303 ± 118
Total proteins (g)	88.1 ± 14.3	100.9 ± 19.6	98.8 ± 11.3	95.9 ± 15.7
Animal protein (g)	56.0 ± 13.8	68.3 ± 20.5	64.8 ± 10.9	63.0 ± 15.7
Plant protein (g)	30.6 ± 4.1	30.9 ± 4.2	32.6 ± 4.0	31.4 ± 4.0
Total lipids (g)	71.2 ± 7.8	75.3 ± 12.5	76.5 ± 12.3	74.3 ± 10.7
SFA (g)	18.3 ± 3.3	19.8 ± 4.6	19.4 ± 4.4	19.1 ± 4.0
MUFA (g)	37.3 ± 5.8	38.0 ± 7.1	40.1 ± 5.8	38.5 ± 6.0
PUFA (g)	7.7 ± 1.6	8.5 ± 2.1	8.7 ± 3.4	8.3 ± 2.4
Total ω-3 (g)	1.4 ± 0.8	1.5 ± 0.9	1.6 ± 0.9	1.5 ± 0.9
Total ω-6 (g)	6.2 ± 1.8	6.7 ± 2.1	7.1 ± 3.0	6.7 ± 3.0
Fibre (g/day)	22.9 ± 4.2	23.2 ± 4.4	24.8 ± 2.9	23.6 ± 3.8
Calcium (mg)	643 ± 254	666 ± 175	638 ± 112	649 ± 180
Iron (mg)	11.9 ± 2.0	14.2 ± 2.9	12.1 ± 1.0	12.7 ± 2.3
Vitamin B12 (mcg)	4.8 ± 2.2	5.3 ± 2.3	6.3 ± 5.1	5.5 ± 3.4
Vitamin C (mg)	225 ± 33	233 ± 28	242 ± 45	233 ± 35
Vitamin E (mg)	13.7 ± 1.9	15 ± 3.2	15.5 ± 2.4	14.8 ± 2.6
Vitamin B1 (mg)	1.4 ± 0.4	1.6 ± 0.4	1.5 ± 0.4	1.5 ± 0.4
Folates (mcg)	342 ± 78	377 ± 138	340 ± 70	353 ± 97
Vitamin B6 (mg)	2.3 ± 0.6	2.7 ± 0.7	2.5 ± 0.4	2.5 ± 0.6

Data represent the daily amounts with the units given in parentheses and are shown as mean ± standard deviation. Data have been calculated through the Metadieta ^®^ software. Data with different letters in the same row are significantly different (*p* < 0.05). CHO, carbohydrates; SFA, saturated fatty acids; MUFA, monounsaturated fatty acids; PUFA, polyunsaturated fatty acids; ω-3, omega-3 fatty acids; ω-6, omega-6 fatty acids.

**Table 3:** Daily mean energy, nutrient and polyphenol intake at baseline in the whole group of subjects, in women and men.

Variables	All (*n* = 51)	Women (*n* = 29)	Men (*n* = 22)	*p*-Value †
Energy (kcal)	1582 ± 108	1569 ± 110	1599 ± 105	0.318
Total CHO (% of energy)	50.0 ± 2.7	50.0 ± 2.7	49.8 ± 2.7	0.641
Simple CHO (% of energy)	20.4 ± 3.1	20.3 ± 3.3	20.5 ± 3.0	0.939
Proteins (% of energy)	17.8 ± 0.8	18.0 ± 0.8	17.7 ± 0.9	0.216
Animal proteins (% of energy)	12.1± 1.1	12.2 ± 1.0	11.8 ± 1.1	0.262
Plant proteins (% of energy)	5.7 ± 0.6	5.7 ± 0.6	5.7 ± 0.6	0.864
Total lipids (% of energy)	32.1 ± 2.3	31.9 ± 2.2	32.4 ± 2.5	0.441
SFA (% of energy)	8.6 ± 1.5	8.6 ± 1.4	8.7 ± 1.7	0.655
MUFA (% of energy)	16.3 ± 1.3	16.3 ± 1.1	16.4 ± 1.6	0.834
PUFA (% of energy)	3.2 ± 0.5	3.3 ± 0.6	3.2 ± 0.4	0.435
ω-3 (% of energy)	0.6 ± 0.2	0.6 ± 0.1	0.6 ± 0.2	0.753
ω-6 (% of energy)	2.5 ± 0.4	2.6 ± 0.5	2.5 ± 0.2	0.341
Total Fibre (g/1000 kcal)	11.2 ± 1.2	11.3 ± 1.1	11.1 ± 1.3	0.458
Cholesterol (mg)	207.7 ± 30.3	204.3 ± 29.9	212.2 ± 30.9	0.682
Total CHO (g)	210.7 ± 21.5	209.6 ± 22.8	212.2 ± 20.1	0.864
Simple CHO (g)	81.0 ± 15.2	80.8 ± 15.4	81.3 ± 15.2	0.954
Proteins (g)	70.3 ± 4.0	70.2 ± 4.0	70.4 ± 4.0	0.849
Animal proteins (g)	47.6 ± 3.9	47.9 ± 3.7	47.2 ± 4.2	0.536
Plant proteins (g)	22.4 ± 2.7	22.2 ± 2.7	22.7 ± 2.8	0.601
Total lipids (g)	56.2 ± 4.9	55.2 ± 3.8	57.5 ± 5.8	0.192
SFA (g)	15.2 ± 3.0	14.9 ± 2.7	15.5 ± 3.3	0.447
MUFA (g)	28.7 ± 2.4	28.3 ± 1.2	29.1 ± 3.4	0.575
PUFA (g)	5.7 ± 0.7	5.7 ± 0.7	5.7 ± 0.8	0.371
Total ω-3 (g)	1.1 ± 0.3	1.1 ± 0.1	1.1 ± 0.4	0.600
Total ω-6 (g)	4.5 ± 0.6	4.5 ± 0.6	4.4 ± 0.5	0.274
Fibre (g/day)	17.8 ± 2.4	17.8 ± 2.2	17.8 ± 2.6	0.932
Calcium (mg)	804 ± 136	808 ± 128	799 ± 147	0.761
Iron (mg)	9.4 ± 0.9	9.4 ± 0.7	9.4 ± 1.0	0.879
Vitamin B12 (µg)	4.2 ± 1.0	4.2 ± 0.4	4.3 ± 1.5	0.394
Vitamin C (mg)	111.8 ± 56.1	115.1 ± 45.2	107.4 ± 68.8	0.464
Vitamin E (mg)	11.4 ± 2.9	11.5 ± 1.3	11.2 ± 4.1	0.327
Vitamin B1 (mg)	0.8 ± 0.2	0.8 ± 0.1	0.7 ± 0.2	0.156
Folates (µg)	302 ± 73	311 ± 54	289 ± 93	0.536
Vitamin B6 (mg)	1.5 ± 0.3	1.5 ± 0.2	1.4 ± 0.3	0.588
Flavonoids (mg)	181.1 ± 137.5	174.4 ± 123.7	190.8 ± 157.8	0.984
Phenolic acids (mg)	130.9 ± 36.0	126.6 ± 28.6	137.1 ± 44.5	0.598
Stilbenes (mg)	0.04 ± 0.06	0.04 ± 0.06	0.04 ± 0.07	0.542
Lignans (mg)	0.8 ± 0.2	0.8 ± 0.2	0.8 ± 0.2	0.737
Other polyphenols (mg)	27.8 ± 4.3	28.0 ± 3.7	27.6 ± 5.0	0.723

All data are presented as mean ± standard deviation (SD); Data with *p* < 0.05 are significantly different. CHO, carbohydrates; SFA, saturated fatty acids; MUFA, monounsaturated fatty acids; PUFA, polyunsaturated fatty acids; ω-3, omega-3 fatty acids; ω-6, omega-6 fatty acids. † Comparison between women and men using Wilcoxon–Mann–Whitney test.

**Table 4:** Intake of total polyphenols and classes (according to PE/USDA databases) during the PR-diet and the C-diet.

Title 1	Flavonoids	Phenolic Acids	Stilbenes	Lignans	Other Polyphenols
**PR-diet**					
All	634.3 ± 171.8	198.1 ± 52.2	0.2 ± 0.4	0.8 ± 0.3	16.4 ± 5.3
Men	594.6 ± 152.2	201.1 ± 74.3	0.4 ± 0.6	0.7 ± 0.3	16.7 ± 6.3
Women	662.1 ± 163.5	195.9 ± 42.0	0.1 ± 0.2	0.8 ± 0.3	16.2 ± 5.3
*p* value #	0.098	0.810	0.108	0.206	0.827
**C-diet**					
All	273.8 ± 119.8	128.2 ± 60.9	0.3 ± 0.5	0.9 ± 0.4	17.0 ± 5.4
Men	260.9 ± 109.6	128.8 ± 57.8	0.4 ± 0.4	0.9 ± 0.4	15.9 ± 5.7
Women	282.9 ± 125.6	127.8 ± 63.0	0.2 ± 0.5	0.9 ± 0.4	17.8 ± 4.9
*p* value #	0.453	0.271	0.033	0.745	0.271
*p* value †	<0.0001	<0.0001	0.386	0.303	0.164
*p* value ¥	<0.0001	0.001	0.575	0.068	0.807
*p* value §	<0.0001	<0.0001	0.348	0.060	0.331

All data are expressed as mean ± standard deviation (SD); PR, polyphenol-rich diet; C, control diet. † Comparison between PR-diet vs. C-diet in women. ¥ Comparison between PR-diet vs. C-diet in men. # Comparison between women and men in PR-diet and C-diet. § Comparison between PR-diet vs. C-diet in all subjects. Comparisons have been performed using the Wilcoxon–Mann–Whitney test.

## Supplementary Materials

The following are available online at https://www.mdpi.com/2072-6643/12/8/2458/s1, Figure S1: comparison of percentage energy and nutrient intake during 8-week polyphenol-rich diet (PR-diet) and control diet (C-diet) in older women and men.
