# Supplementary material for: Estimated Intakes of Nutrients and Polyphenols in Participants Completing the MaPLE Randomised Controlled Trial and Its Relevance for the Future Development of Dietary Guidelines for the Older Subjects
Source: Nutrients. 2020 Aug 15;12(8):2458. doi: 10.3390/nu12082458 (PMC7468770; doi:10.3390/nu12082458)
Supplement: Supplementary file 1 [file nutrients-12-02458-s001.zip › nutrients-898100-supplementary.docx]

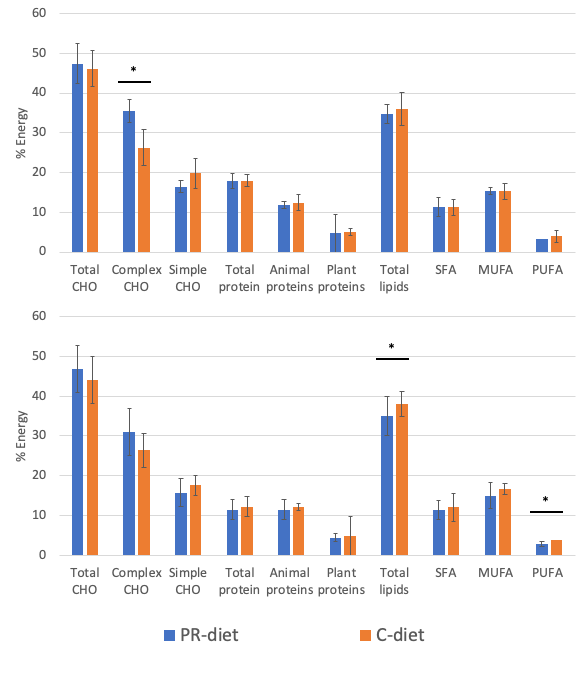


**Suppl. Figure S1** **Comparison of percentage energy and nutrient intake during 8-week polyphenol-rich diet (PR-diet) and control diet (C-diet) in older women and men.**

Data are expressed as mean ± standard deviation (SD); Data with asterisks are significantly different (P<0.05). Comparisons were made by using Wilcoxon-Mann-Whitney test.

Legend: PR, polyphenol-rich diet; C, control diet; CHO, carbohydrates; SFA, saturated fatty acids; MUFA, monounsaturated fatty acids; PUFA, polyunsaturated fatty acids.
